# Analysis of the Part Distortions for Inconel 718 SLM: A Case Study on the NIST Test Artifact

**DOI:** 10.3390/ma13225087

**Published:** 2020-11-11

**Authors:** Silvia Martínez, Naiara Ortega, Diego Celentano, Antonio J. Sánchez Egea, Eneko Ukar, A. Lamikiz

**Affiliations:** 1 Department of Mechanical Engineering, Aeronautics Advanced Manufacturing Center (CFAA), Faculty of Engineering of Bilbao, Alameda de Urquijo s/n, 48013 Bilbao, Spain; silvia.martinez@ehu.eus (S.M.); naiara.ortega@ehu.es (N.O.); eneko.ukar@ehu.es (E.U.); 2 Department of Mechanical and Metallurgical Engineering, Pontificia Universidad Católica de Chile, 782-0436 Región Metropolitana, Chile; dcelentano@ing.puc.cl; 3 Department of Mechanical Engineering (EEBE), Universitat Politècnica de Catalunya, Av. D’Eduard Maristany, 16, 08019 Barcelona, Spain; antonio.egea@upc.edu

**Keywords:** SLM, geometry distortion, Inconel 718, substrate thickness, numerical simulation

## Abstract

The present paper evaluates the misalignment and geometry distortion of the standard National Institute of Standards and Technology (NIST) test artifact in Inconel 718 alloy, when several layers with and without supports are employed to manufacture it by the Selective Laser Melting (SLM) process. To this end, a coordinate-measuring machine (CMM) is used to measure the geometrical distortion in each manufacturing configuration, following the same measurement protocol. The results show that the laser path strategy favors a thermal gradient which, consequently, induces geometrical distortions in the part. To prove this hypothesis, a numerical simulation is performed to determine the thermal gradient and the pattern of the residual stresses. It was found that the geometrical distortion certainly depends on the position of the feature position and laser strategy, where thermal cycles and residual thermal stresses had an impact in the end-part geometry, especially if a high strength-to-weight ratio commonly used in aeronautics is present.

## 1. Introduction

Metal Selective Laser Melting (SLM) is currently one of the most relevant manufacturing techniques and is presently considered to be a hot-topic in mechanical and material science research areas [1]. The impact of this process relies on the good acceptance in highly regulated industries such as aerospace and medical devices. However, in additive manufacturing (AM) processes undesired geometry distortions and shrinkages are the crucial problems to overcome [2]. As a result, much literature was focused on addressing an effective way to quantify and, ultimately, minimize these geometrical issues. Novel approaches used calibrated analytical thermal models to predict with finite element methods the distortion of the material geometry due to thermal stress [3,4]. Numerical models that took into account indirect coupled thermomechanical effects characterized the structural variability on a single layer of a thin-walled of Ti64 part due to the temperature gradients and thermal stresses that develop in the material when using different scan lengths [5]. Similar experiments were carried out to address geometry distortion of the substrate during build-up a specimen by using in situ laser distance measurements [6]. Regarding the measurement techniques, Scaravetti et al. [7] described a method to identify if the geometrical defects came from either the process or the specimen distortion while it is manufactured. However, they did not come with a straight forward solution, because a modification of the operational parameters of the machine is required that in some cases will affect the material’s behavior. Recently, Tan et al. [8] focused on optimizing the performance of SLM to obtain end products by understanding the issues of different powder characteristics, such us recycled powder (from unfused particles). They found that packing density and flowability are influenced by the powder size deviation which noticeably affects the efficiency of the SLM process. Besides, the thermal cycles have been experimentally studied to determine the material microstructure of Inconel 718 manufactured by SLM [9,10], promoting the material texture and high misorientation angles, whereas the grains tend to coarse when longer thermal cycles are performed.

Despite the fact that a large number of standardization of methods were reported to achieve an effective additively manufactured part production, it is still required to improve the process disparities via monitoring and measuring tasks in order to ensure the industry compliances of quality. Initially, Campanelli et al. [11] proposed a model to statistically measure the main geometrical distortions of built parts with the stereolithographic technology. However, the most renowned test artifact to evaluate the manufacturing quality in AM is the test workpiece designed by Moylan et al. [12], which in collaboration with the National Institute of Standards and Technology (NIST) [13], is presently widely accepted as the standardization test artifact in the AM field. Regarding the measurement tasks, it is really challenging to predict the geometrical distortion in specimens manufactured by SLM. These distortions are mainly attributed to the microstructure change due to the rapid heat treatment and large non-uniform thermal gradients which as a consequence, induce thermal stresses and volumetric contraction (shrinkage) that change the melted material and its anisotropic properties [14]. According to this, several shrinkage calibration tests were performed with the aim of optimizing the selective laser sintering process [15]. As expected, the sintering strategy and the part orientation to the beam had an influence in the final accuracy of the end product, while the beam offset and a proper hatching configuration minimize geometrical errors [16]. Additionally, the residual stresses also affect the final accuracy of the specimen, particularly if any annealing thermal treatment is performed. Recently, Li et al. [17] proposed a prediction model to estimate the distortion of the specimen manufactured by SLM based on the residual stresses induced during this manufacturing process. The results exhibited different cantilever distortions according to the specimen thickness where, in addition, the residual stresses were numerically estimated before and after the support structure was cut from the substrate. It was concluded that the part distortion due to residual stress depends on the material thickness, the temperature history, and the melting configuration. They proved that 70% of the residual stresses were found to be released when removing the supports from the specimen. Following this last research topic, the present study focuses on evaluating the misalignment and geometry distortion of the NIST test artifact in Inconel 718 alloy. To achieve that, several layers with and without supports are employed to manufacture the NIST workpiece with the SLM process, while using the same manufacturing configuration and workpiece orientation. Finally, numerical simulations are carried out to assess how the residual stresses affect the manufacturing accuracy when considering SLM test artifact under different support and subtract configurations.

## 2. Materials and Methods

**Figure 1:**
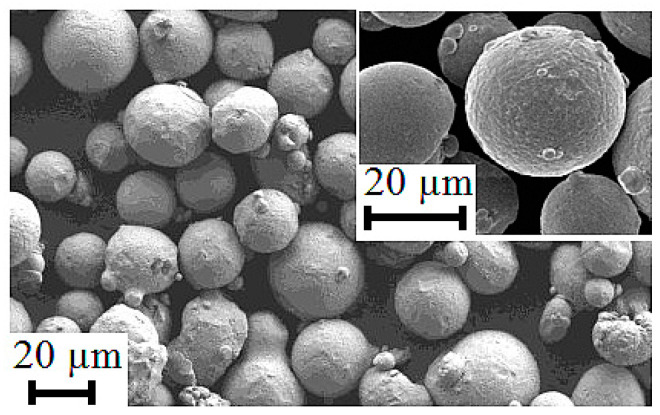
SEM images of the morphology of the Inconel 718 powder.

The SLM manufacturing process has been carried out with a Renishaw AM400 machine (Machineseeker, UK). This additive manufacturing machine disposes 250 mm × 250 mm × 300 mm for the x, y and z axis, respectively. The operational parameters used in the SLMed NIST part were: 70 μm focal spot size, 40 μs exposure time, 30 μm layer thickness, 200 W power, 35 μm hatching distance, 1.75 m/s scanning speed and, consequently, 108.84 J/mm^3^ laser energy density [18]. The manufacturing protocol of the test artifact is as follows: firstly, it is designed the computer-aided design (CAD) geometry, converted into a stereolithography (STL) file and divided into layers of 30 μm of thickness where, in addition, the chosen strategy consists of 10 mm width stripes with a difference of 67° between each layer. Then, the additive strategy and process parameters depending on the part material and its geometric features, such as internal features and contour zones, were selected. Later, the powder deposition, the laser spot guided by a galvanometric head, and the scanner melts the part zone with a stripe trajectory were defined. Once the layer has been processed, the substrate is moved down up to a distance corresponding to the layer thickness and then another new layer of powder is deposited and extended by the tracker to onset the additive manufacturing process of the second layer. The powder material used is Nickel Alloy Powder (REN-in718) with particle sizes between 15–45 µm. Table 1 lists the chemical composition of the used powder. Figure 1 exhibits the SEM images of the morphology of the Inconel 718 powder.

**Figure 2:**
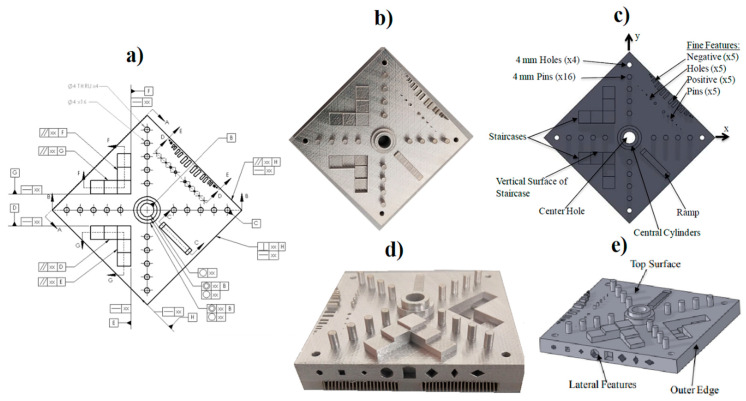
Supported and non-supported NIST test artifact, technical drawing and 3D model of the test artifact used in the SLM and coordinate-measuring machines [13]. (**a**) exhibits the technical drawing and dimensional tolerances. (**b**,**d**) show the supported NIST test artifact and (**c**,**e**) exhibit the main features of the 3D designed NIST test artifact.

The manufactured test piece to evaluate the geometrical distortion was developed by the National Institute of Standards and Technology (NIST) for standardization purposes. Figure 2 shows the overview and the technical drawing of the manufactured NIST artifact. 

**Figure 3:**
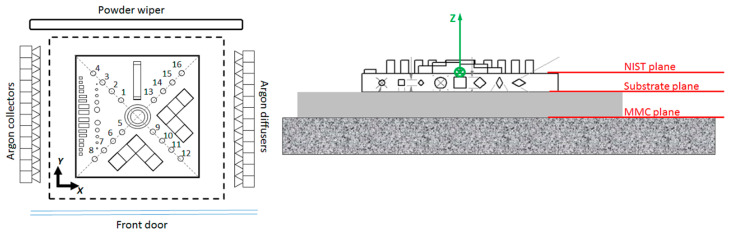
SLM workpiece orientation and CMM configurations and used to analyze the geometrical distortions of the SLMed NIST part in Inconel 718 alloy.

The geometrical measurements have been carried out with a MITUTOYO Crysta Apex S 9106 CMM (Neuss, Germany), using two strategies: point to point and scanning touch probes (probe Φ 3 mm). The obtained results show that the maximum accuracy of this process (under these conditions and parameters) is not better than 0.175 mm. Figure 3 exhibits the reference planes used in the coordinate-measuring machine (CMM) to analyze the geometrical distortions of the SLMed NIST part in Inconel 718 alloy. 

## 3. Results

**Figure 4:**
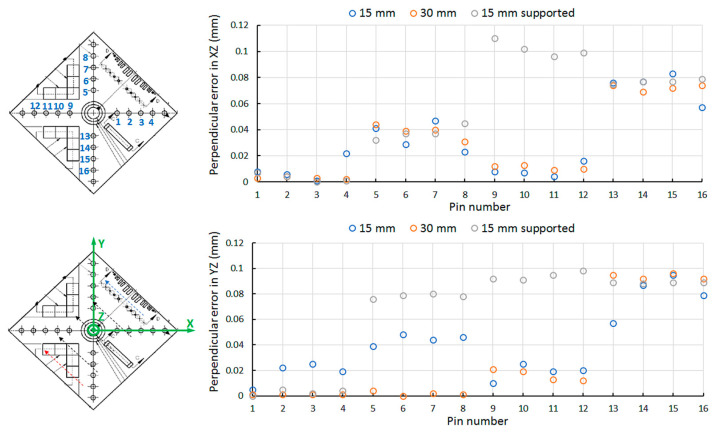
Geometrical distortion of each pin number with respect to the XZ and YZ planes.

The orientation of the features that defines the final sample within the AM device and laser path strategy will have an influence on the final part geometry due to the thermal gradients occurring during the manufacturing process. These thermal gradients are associated with the laser paths and the locations where the material melting occurs. To determine the influence of these thermal gradients on the geometrical distortion of ends parts, the NIST part is SLM printed under three configurations: 15 mm thickness of substrate, 30 mm thickness of substrate and 15 mm thickness of substrate and supports between the part and the substrate. Accordingly, Figure 4 shows the geometrical distortions of 16 pins in the XZ and YZ planes. Thus, this figure exhibits the perpendicular error of the pins with respect to the predefined X and Y directions.

**Figure 5:**
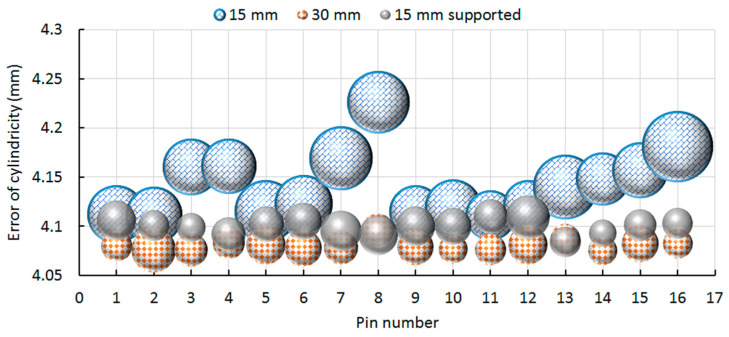
Error of cylindricity for the 16 pins allocated on top of the NIST part.

These geometrical results show that the four pins where the laser begins to melt material do not present a high perpendicular error in the XZ plane with only 0.02 mm of deviation toward the YZ plane. On the contrary, larger perpendicular errors are found for the pins allocated in the negative X axis with an error dispersion of 0.08 and 0.1 mm in both planes, independently of the substrate configuration. Looking at the supported configuration, the error dispersion is noticeably high in the pins independently of the situation. It seems that the supports are not helping to reduce the geometrical distortion associated with the thermal gradients. Figure 5 shows the cylindricity error for the 16 pins. It is denoted that the substrate with 15 mm of thickness exhibits larger magnitude of error and margin errors, noted as the diameter of the circles. No significant differences between the substrate with 30 mm of thickness and the supported substrate with 15 mm of thickness are appreciated, despite that lower average values are found for the thicker substrate and lower margin errors are observed in the supported substrate.

**Figure 6:**
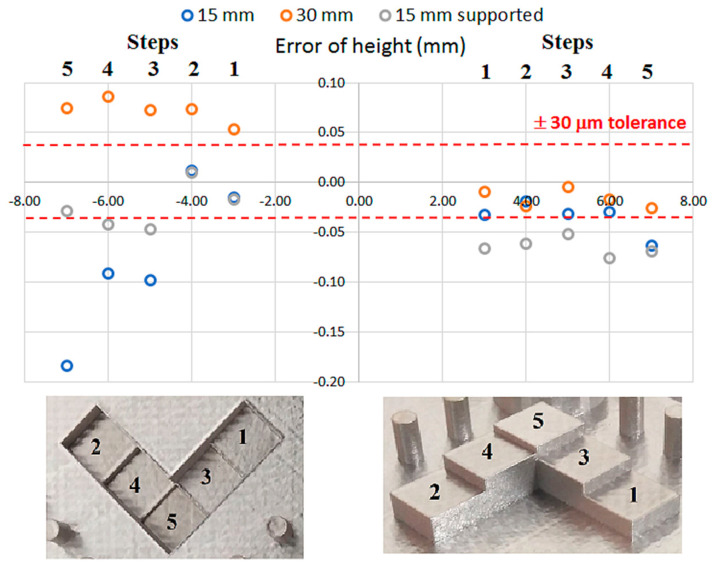
Error of height for the SLMed stairs that go up and down in the NIST part.

Looking to the results found for the 15 mm thickness substrate, the y-axis induces higher distortion in the cylindricity of the features. A smaller error is found when the feature is allocated near the center of the NIST part and a larger error is found in the distal position where in particular, error values about 0.20–0.25 mm are found for pin 8 and 16. It can be also noticed that the cylindricity error increases as we move away from the center of the NIST part. It can be mentioned that the 8 and 16 pin which are allocated in the opposite corner present the larger cylindricity error that could be attributed to the thermal gradient associated with the laser trajectory and, subsequently, to dissimilar substrate deformation. Moreover, Figure 6 shows the height error for the stairs that go up and down in the NIST part.

The measurement of the stair position is divided in two, the stair that goes down (represented in the left part of Figure 5) and the star that goes up (represented in the right part of Figure 5). The stair that goes down shows the largest error of height, where negative error values of about −0.20 mm are observed for the 15 mm thickness substrate. Larger errors also occur for the first three steps which are the closer to the substrate, while the last two steps are within the acceptable tolerance of ±30 µm. On the contrary, the 30 mm thickness substrate exhibits a common positive error of about 0.05 and 0.1 mm for all the steps of the stair, which seems to be an offset that had influenced all the steps of the stair. Finally, the 15 mm thickness and supported substrate denotes the smaller error for this type of stair. Looking at the error values of the stair that goes up, both the 15 mm and 30 thickness substrates present similar error values within the tolerance of ±30 µm, except for the last step of the thinner substrate that goes beyond −0.05 mm. The supported substrate also shows stable results but out of the desired range. All the values are found to be around −0.05 mm and no difference is found for the position of the step.

## 4. Discussions

The experimental results presented above need to be explained through an analysis of the thermomechanical response of the Inconel 718 substrate during the manufacturing process. This task is tackled in this work via a numerical simulation of a representative Inconel 718 6-layer deposition sequence with the aim of providing indications of why the observed geometrical distortions are developed in the NIST part. In this context, the numerical results can only be considered to be qualitative, a fact that prevents their subsequent quantitative experimental validation. Square layers of 10 mm side and 30 µm thickness are deposited in the center of a square substrate of 30 mm side and 15 mm high. The operating parameters are those selected for the manufacturing of the NIST artifact, i.e., laser beam diameter, power and scanning velocity of 70 µm, 200 W and 1.75 m/s, respectively, considering and overlapping between successive scan paths of 50% (i.e., 35 μm of hatching distance) and an absorptivity of 0.6 [19]. The time elapsed between layers is 5 s. The substrate is clamped in its base throughout the process. The initial temperature of both the substrate and the layers is equal to the environmental temperature (25 °C). The numerical simulation of the layer deposition process followed by a cooling stage is carried out in the context of the finite element method via a coupled thermomechanical formulation which encompasses the energy and momentum balance equations [20]. The Inconel 718 thermomechanical properties used in the computations are those reported in Promoppatum et al. [19] and references therein, where temperature-dependent values defined for the powder, solid and liquid phases are defined. Latent heat absorption/release effects due to solid-liquid phase-change are also included. An elastoplastic stress-strain law is taken into account for describing the material mechanical response for all these phases. In particular, the deviatoric/volumetric decomposition of the elastic isotropic constitutive tensor proposed by Celentano et al. [20] is chosen here in order to obtain in the liquid phase a purely elastic volumetric behavior, i.e., only pressure without shear stresses can develop in such phase. Each layer is spatially discretized with 22,500 8-noded elements, such that the element side length in the irradiation plane is slightly smaller than the laser beam diameter in order to properly capture the high expected temperature gradients expected in the vicinity of the laser path. The manufacturing sequence is simulated by the standard element activation technique. The time is discretized with a time step of 2.5 × 10^−4^ s which corresponds, for numerical accuracy reasons, to nearly 70% of the time needed for the laser beam to cover a distance equal to its diameter.

**Figure 7:**
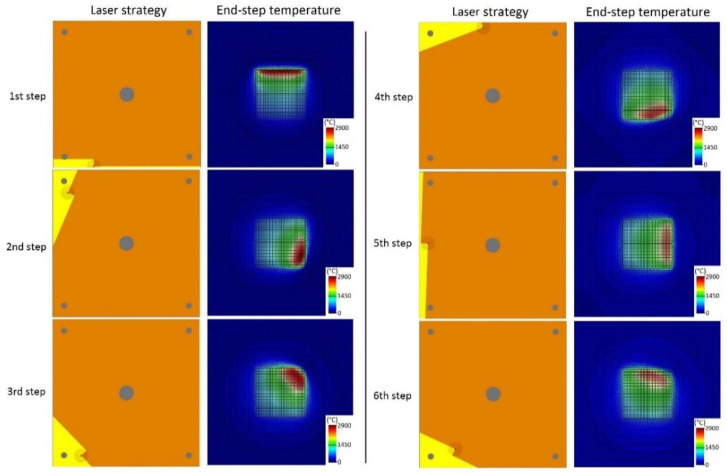
Scanning sequence and computed temperature contours of the substrate surface at the end of each layer step of the SLMed NIST part in Inconel 718 alloy.

**Figure 8:**
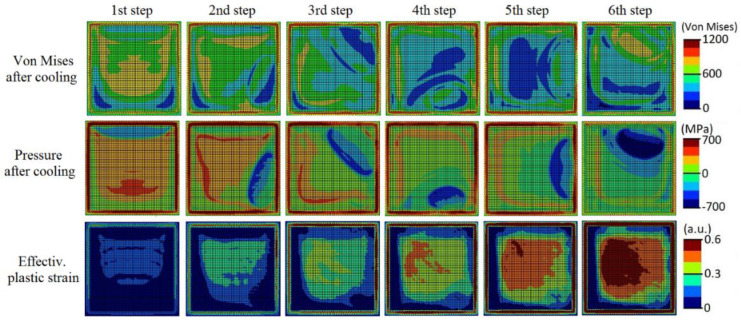
Computed residual von Mises stress, pressure, and effective plastic strain contours of the substrate surface at the end of the cooling stage of each layer step of the SLMed NIST part.

Figure 7 depicts the Inconel 718 SLM scanning sequence of the six layers together with the temperature contours of the substrate surface at the end of each layer step while Figure 8 shows the residual von Mises stress, pressure and effective plastic strain contours of the substrate surface at the end of the cooling stage of each layer step (i.e., after 5 s of the final instant of the deposition of each layer). It is seen that the temperature at the substrate surface is very high, i.e., higher than the melting point. As already reported in Stevens et al. [21], it is seen that highly localized temperature gradients are developed during the whole deposition sequence. The temperature contours also exhibit values well beyond the melting temperature. Consequently, a small liquid region is produced in which as mentioned above, the material only reacts with a negative pressure field due to thermal dilatation prevented by the colder surrounding material. However, relatively high stress values (the material yield strength at 25 °C is 1375 MPa) with a non-uniform distribution, which spatially varies during the deposition sequence, are generated at the end of the cooling stage of each layer step. Although the tension pressure patterns show a diminishing trend evolution probably due to the more uniform temperature gradients caused by thermal conduction in the substrate as the layers are generated, the peak values of the von Mises stress remain nearly the same along the process. Moreover, as expected, the continuously increasing levels of accumulated effective plastic strain could be the cause that leads to geometric distortions of the final piece [22]. 

## 5. Conclusions

The present paper successfully evaluates the misalignment and geometry distortion of the NIST test artifact in Inconel 718 alloy associated with different substrates configurations and for a particular laser trajectory. To this end, the following findings can be drawn:

- The experimental results showed different geometrical distortions in several features of the SLMed NIST part, where using 15 mm thickness of substrate, 30 mm thickness of substrate and 15 mm thickness of substrate and supports between the part and the substrate. In general, a thicker substrate denoted lower distortion, but a thin substrate combined with supports has found to be an acceptable solution, except for perpendicularity of the pins in the YZ plane
- The thermomechanical simulation has proved the occurrence of high thermal gradients after each layer deposition of the SLM. These thermal gradients leaded to thermal residual stresses at the substrate that have been at least one of the main factor to the geometrical distortion found in the SLMed NIST part.
- Based on the experimental and simulation results, the laser path strategy and substrate thickness are crucial parameters to take into account when SLM is performed on large areas. It is found that high thermal gradients promote residual stresses at the substrate that may have an impact on the dimensional tolerance of the printed end-parts.

In future work, it is planned to manufacture cantilevers with different thickness by using SLM with the aim to experimentally and numerically analyze their residual stresses. In this sense, we will study the influence of the laser strategy on the cantilever thickness and, at the same time, improve the robustness of the numerical model according to the deformation of the workpiece after been detached.

## Figures and Tables

**Table 1:** Material chemical composition by percent of weight of Inconel 718.

%Ni	%Cr	%Nb	%Mo	%Ti	%Al	%C	%Si	%Co	%Fe
50–55	17–21	4.8–5.5	2.8-3.3	0.8–1.2	0.3–0.7	0.02–0.08	<0.35	<1.00	Balance
